# Insights into the Quorum Sensing Regulon of the Acidophilic *Acidithiobacillus ferrooxidans* Revealed by Transcriptomic in the Presence of an Acyl Homoserine Lactone Superagonist Analog

**DOI:** 10.3389/fmicb.2016.01365

**Published:** 2016-09-14

**Authors:** Sigde Mamani, Danielle Moinier, Yann Denis, Laurent Soulère, Yves Queneau, Emmanuel Talla, Violaine Bonnefoy, Nicolas Guiliani

**Affiliations:** ^1^Laboratoire de Chimie Bactérienne, Institut de Microbiologie de la Méditerranée, Aix Marseille Université, Centre National de la Recherche ScientifiqueMarseille, France; ^2^Laboratorio de Comunicación Bacteriana, Departamento de Biología, Facultad de Ciencias, Universitad de ChileSantiago, Chile; ^3^Plateforme Transcriptome, Institut de Microbiologie de la Méditerranée, Aix Marseille Université, Centre National de la Recherche ScientifiqueMarseille, France; ^4^Université Lyon, Institut National des Sciences Appliquées de Lyon, UMR 5246, Centre National de la Recherche Scientifique, Université Lyon 1, École Supérieure de Chimie Physique Electronique de Lyon, Institut de Chimie et de Biochimie Moléculaires et SupramoléculairesVilleurbanne, France

**Keywords:** quorum sensing regulon, acyl homoserine lactone, superagonist, extracellular polymeric substances, biofilm, transcriptomic, *Acidithiobacillus ferrooxidans*, acidophile

## Abstract

While a functional quorum sensing system has been identified in the acidophilic chemolithoautotrophic *Acidithiobacillus ferrooxidans* ATCC 23270^T^ and shown to modulate cell adhesion to solid substrates, nothing is known about the genes it regulates. To address the question of how quorum sensing controls biofilm formation in *A*. *ferrooxidans*^T^, the transcriptome of this organism in conditions in which quorum sensing response is stimulated by a synthetic superagonist AHL (N-acyl homoserine lactones) analog has been studied. First, the effect on biofilm formation of a synthetic AHL tetrazolic analog, tetrazole **9c**, known for its agonistic QS activity, was assessed by fluorescence and electron microscopy. A fast adherence of *A. ferrooxidans*^T^ cells on sulfur coupons was observed. Then, tetrazole **9c** was used in DNA microarray experiments that allowed the identification of genes regulated by quorum sensing signaling, and more particularly, those involved in early biofilm formation. Interestingly, *afeI* gene, encoding the AHL synthase, but not the *A. ferrooxidans* quorum sensing transcriptional regulator AfeR encoding gene, was shown to be regulated by quorum sensing. Data indicated that quorum sensing network represents at least 4.5% (141 genes) of the ATCC 23270^T^ genome of which 42.5% (60 genes) are related to biofilm formation. Finally, AfeR was shown to bind specifically to the regulatory region of the *afeI* gene at the level of the palindromic sequence predicted to be the AfeR binding site. Our results give new insights on the response of *A. ferrooxidans* to quorum sensing and on biofilm biogenesis.

## Introduction

Due to its low operating cost, biomining is a very successful geobiotechnology that actually produces approximately 15 per cent of the world’s extracted copper (Johnson, 2014). Withstanding low pH and high heavy metal concentrations, *Acidithiobacillus* species are acidophilic key players in biomining industry recovering valuable metals from sulfidic ores such as copper or gold (Jerez, 2009). However, these bacteria are also involved in Acid Mine/Rock Drainage (AM/RD), which represents a worldwide problem of water pollution, from natural and anthropogenic environments (Johnson, 2009, 2012). Indeed, several studies recently indicated that *Acidithiobacillus* species play a pivotal and structural role in acidophilic communities ranging from 6°C to 90°C (Chen et al., 2015; Liljeqvist et al., 2015; Menzel et al., 2015). Nevertheless, due to an insufficient understanding of the microbiological processes, most biohydrometallurgical plants operate far from maximum efficiency and natural AM/RD are to a large extent uncontrolled.

Acidithiobacillia has been recently defined as a new class of *Proteobacteria* in which the genus *Acidithiobacillus* is the main one characterized (Williams and Kelly, 2013). Actually, the genus *Acidithiobacillus* encompasses seven closely related Gram-negative, chemolithoautotrophic bioleaching species: (i) *Acidithiobacillus thiooxidans, A. caldus*, and *A. albertensis*, which oxidize only reduced inorganic sulfur compounds (RISC) and (ii) *A ferrooxidans, A. ferrivorans, A. ferridurans*, and *A. ferriphilus* that oxidize both ferrous iron and RISC (Amouric et al., 2011; Hedrich and Johnson, 2013; Williams and Kelly, 2013; Falagan and Johnson, 2015). It has been well established that all *Acidithiobacillus* species are able to form biofilms on the surface of ores. This bacterial attachment on the mineral has been reported to increase metal leaching due to the formation of a close and enlarged “reaction space” between the metal sulfide surface and the cell (Pogliani and Donati, 1999; Harneit et al., 2006; Rohwerder and Sand, 2007). Therefore, deciphering molecular mechanisms underlying biofilm formation in acidophilic leaching bacteria has been early pointed out as an important field of investigation.

Quorum sensing (QS) and the secondary messenger c-di-GMP signaling pathway [for recent reviews see (Hengge, 2009; Decho et al., 2011; Kalia et al., 2013; Romling et al., 2013; Hengge et al., 2015)] are the most studied mechanisms controlling biofilm development in bacteria. Both pathways have been shown to be linked in several bacterial species (Ryan et al., 2006; Waters et al., 2008; Ueda and Wood, 2009; Zhang, 2010; Kozlova et al., 2011) and to control more particularly polysaccharide production and biofilm formation (Ueda and Wood, 2009). QS is an important mechanism for the timing of collective behaviors through the regulation of population density-dependent cellular processes, such as the production of virulence factors, motility, exopolysaccharide production and biofilm formation (Parsek and Greenberg, 2005; Waters and Bassler, 2005; Ng and Bassler, 2009). In Gram-negative bacteria, the main characterized QS system involves three key molecular elements (Venturi and Subramoni, 2009): (i) N-acyl homoserine lactones (AHLs), which act as autoinducers (AIs); (ii) the AHLs synthase encoded by a *luxI*-like gene; (iii) a transcriptional regulator, which is encoded by a *luxR*-like gene and which binds AHL molecules and modulates the expression of different target genes that constitute the QS regulon. Depending on the bacterial species and also on the experimental strategies (transcriptomic or proteomic), the size of the QS regulons oscillates between 3 and 8% of the identified ORFs (Vasil, 2003; Wagner et al., 2003; Cantero et al., 2006; Qin et al., 2007; Stevens et al., 2011; Majerczyk et al., 2014).

Even if several reports related to biofilm formation regulation by acidophilic bacteria belonging to *Acidithiobacillus* genus have been released recently (Farah et al., 2005; Bellenberg et al., 2012, 2014; Ruiz et al., 2012; Diaz et al., 2013; Montgomery et al., 2013; Vera et al., 2013; Castro et al., 2015), the molecular cascade involved in exopolysaccharide production and biofilm formation by *Acidithiobacillus* species is still undeciphered. While c-di-GMP pathway has been identified in all *Acidithiobacillus* spp. (Ruiz et al., 2012; Diaz et al., 2013; Castro et al., 2015), the species that oxidize only RISC do not possess the genes related to canonical QS systems (Valdés et al., 2008). Indeed, a functional QS system has been reported only in the iron/RISC-oxidizing species *A. ferrooxidans* (Farah et al., 2005; Rivas et al., 2005; Valenzuela et al., 2007). In addition, it has been recently reported that the RISC-oxidizing species *A. thiooxidans* cannot adhere to pyrite if this mineral is not previously colonized by an iron-oxidizing species (Bellenberg et al., 2014) pointing out *A. ferrooxidans* as a key player for mineral colonization.

*Acidithiobacillus ferrooxidans* ATCC 23270^T^ QS system involves two divergent genes *afeI* and *afeR* coding for the AHL synthase and the transcriptional regulator, respectively (Farah et al., 2005). AfeR has the conserved amino acid residues located in the active site of LuxR-protein family and possesses the canonical AHL and DNA binding domains based on a 3D-structural model (Soulere et al., 2008). In *A. ferrooxidans* ATCC 23270^T^, nine different AHL molecules are synthesized with medium or large acyl side chains (Valenzuela et al., 2007). In this strain, transcription of *afeI* is increased under the physiological conditions that promote biofilm formation, such as growth in the presence of sulfur (solid energetic substrate) or in low phosphate medium (Farah et al., 2005), suggesting a role of QS system in the attachment of *A. ferrooxidans* to ores (e.g., pyrite). In agreement with this hypothesis, addition of synthetic AHL that are AIs naturally synthesized by *A. ferrooxidans* such as C14-AHL and 3-hydroxy-C14-AHL has been shown to enhance *A. ferrooxidans* ATCC 23270^T^ cell adhesion, exopolysaccharide production and biofilm development on elemental sulfur and pyrite (Ruiz et al., 2008; Gonzalez et al., 2013).

However, to date this phenotypic result is still uncoupled with genotypic data that will allow the understanding of the molecular chain reaction going from the AHL-sensing by AfeR to ore colonization. A bioinformatics analysis has recently allowed the identification of a putative QS regulon in *A. ferrooxidans* ATCC 23270^T^ that encompasses 75 possible AfeR target-genes, including genes likely involved in polysaccharide biosynthesis (Banderas and Guiliani, 2013). However, biological data are required to fully identify the *A. ferrooxidans* genes whose expression is modulated by AHL signaling.

Here, we report the first biological study focused on deciphering the QS regulon of *A. ferrooxidans* ATCC 23270^T^. The effects of AI 3-hydroxy-C14-AHL and of tetrazolic AHL-analog **9c**, on *A. ferrooxidans* adhesion to sulfur were first compared by fluorescence and scanning electronic microscopy. Then, DNA microarray experiments were performed to compare total RNA of *A. ferrooxidans* ATCC 23270^T^ cells induced or not by tetrazole **9c**. These allowed the identification of 141 genes from which at least 48 can be linked with QS pathway, exopolysaccharide production and biofilm development. If we include the genes encoding hypothetical proteins that colocalized and are coregulated with these 48 genes, this number would increase to 60 and represents 1.9% of the ATCC 23270^T^ genome.

## Materials and Methods

### Bacterial Strains, Plasmids, and Growth Conditions

*Acidithiobacillus ferrooxidans* ATCC 23270^T^ was used throughout this study. *Escherichia coli* TG1 [(*supE, hsd*Δ5, *thi*, Δ (*lac-proAB*), F’:*traD36, proAB^+^, lacI^q^, lacZ*ΔM15) was used for plasmid propagation. Rosetta (DE3)/pLysS strain (F^-^ *ompT hsd*S_B_(*r_B_*^-^ *m_B_*^-^) *gal dcm* λ [DE3 (*lacI lacUV5*-T7 gene *1 ind1 sam7 nin5*)] pLysSRARE (Cam^R^)] and the pET21 plasmid from Novagen were used to produce the recombinant AfeR with a hexahistidine tag fused to its C terminus (AfeR-Histag).

*Acidithiobacillus ferrooxidans* was grown at 30°C under oxic conditions in modified 9K medium [(0.1 g L^-1^ NH_4_)_2_SO_4_, 0.4 g L^-1^ MgSO_4_⋅7H_2_O; 0.04 g L^-1^ K_2_HPO_4_, pH 2,5] with sulfur (S^0^) coupons (0.5 cm^2^ obtained by S^0^ fusion) for fluorescence and electron microscopy or 200 g L^-1^ S^0^ prills for real-time PCR or microarrays analysis (Amaro et al., 1991) in the presence (5 μM) or the absence of the AHL analogs. The ferrous iron [Fe(II)] growth conditions were described in (Yarzabal et al., 2003). *E. coli* strains were usually grown at 37°C under oxic conditions in Luria-Bertani broth (LB) supplemented with 100 μg ml^-1^ ampicillin and 34 μg ml^-1^ chloramphenicol when necessary (Ausubel et al., 1998).

### Synthesis of AHL-Signaling Molecules

Due to its high agonistic effect reported on *Vibrio fischeri* QS system (Sabbah et al., 2012), the tetrazolic AHL analog (tetrazole **9c**; **Supplementary Figure S1**) was selected to test its biological activity on biofilm formation by *A. ferrooxidans*. It was synthesized according to the protocol described by Sabbah et al. (2012). Briefly, this synthesis was achieved from racemic α-amino-γ-butyrolactone hydrobromide that was acylated with heptanoyl chloride. The intermediate was then cyclized with sodium azide (Biot et al., 2004) to afford tetrazole **9c** (**Supplementary Figure S1**). *A. ferrooxidans* natural AI 3-hydroxy-C14-AHL was also obtained by chemical synthesis according to the protocol described previously (Chhabra et al., 2003).

### Cell Adhesion Assays on Sulfur Coupons and Microscopy Visualizations

Experimental procedures have been previously described (Gonzalez et al., 2013). *A*. *ferrooxidans* was grown at 30°C in modified 9K medium (Ruiz et al., 2012) at pH 2.5 with 5% (wt/vol) sulfur (S^0^) prills. To assess adhesion levels, sterilized S^0^ coupons were initially added to cell cultures. S^0^ coupons were daily extracted from day 1 (lag phase) to day 7 (end of the exponential phase) and adhered cells were fixed. Staining was performed with fluorochrome Syto9 for epifluorescence microscopy observations. Epifluorescence visualizations of stained coupons were performed by using fluorescence microscope (ZEISS Axiovert 200 M) equipped with a filter set 10 (FITC, emission BP 515–565) and 20 (Rhodamine, emission BP 575–640) and a digital microscope camera (Axiocam ZEISS). For scanning electronic microscopy (SEM) visualizations, S^0^ coupons colonized by *A. ferrooxidans* cells were submitted to critical point drying to avoid cell shrinking and damage. Then, dried samples were coated with a thin conductive film of gold and analyzed with a scanning electron microscope (HITACHI TM 3000, Japan) at the Pontificia Universidad Católica de Chile.

### General DNA Manipulations

Genomic DNA from *A. ferrooxidans* was prepared with the NucleoSpin Tissue kit (Macherey Nagel). Plasmid DNA was obtained using a Wizard Plus SV DNA purification system from Promega. DNA digestions with restriction enzymes and ligation with T4 DNA ligase were performed according to New England BioLabs’ recommendations. Primers (Sigma) used in this study are described in Supplementary Table S1. For routine PCR, Go *Taq* polymerase (Promega) was used. For *afeR* cloning, PCR amplifications were carried out with Platinum *Taq* polymerase (Invitrogen) on genomic DNA. DNA products were analyzed on an 1% agarose gel, then concentrated and purified using Amicon^®^ Ultra-0.5 centrifugal filter units (Millipore). Recombinant plasmids were introduced into *E. coli* competent cells as previously described (Chung and Miller, 1988).

Nucleotide sequence of the amplified DNA was determined by GATC Biotech (Germany).

### RNA Manipulations

To get reproducible results, the following experimental growth protocol was performed. The starting inoculum was obtained by growing 1 × 10^7^ cells on 150 ml Fe (II) medium for 3 days. From this culture, 1 × 10^7^ cells were washed three times with basal salts to remove iron traces and inoculated in 250 ml 9K modified medium containing 200 g L^-1^ S^0^ prills for 5–6 days (adaptation step). This culture was used to inoculate the same medium (400 ml) for 4 days (pre-inoculum step). This step was repeated in larger volumes in the presence of superagonist AHL analog (adding 5 μM tetrazole **9c**) and in its absence (adding DMSO which is the tetrazole **9c** solvent) and the cultures were grown for 2, 3, and 4 days.

The cultures were centrifuged at low speed (1,000 rpm, 5 min) to recover S^0^ prills. Planktonic cells were harvested from the supernatant by centrifugation and washed several times with acid water (pH 1.5) to remove S^0^. To get sessile cells, the collected S^0^ prills were washed several times with acid water to remove the remaining planktonic cells. Then, S^0^ prills were incubated for 5 min in acid water with 0.04% Triton X-100. They were vortexed every min and then, sonicated every 4 sec for 2 min at 4°C to recover adhered cells. S^0^ prills were removed by low speed centrifugation (1,000 rpm, 5 min). Sessile cells, harvested by centrifugation from the supernatant, were washed three times with acid water to remove Triton X-100.

*Acidithiobacillus. ferrooxidans* total RNA was extracted from planktonic and sessile cells by using a modified acid-phenol extraction method (Aiba et al., 1981) according to Quatrini et al. (2006, 2009). The modifications included a preliminary TRIZOL^®^ reagent (Invitrogen) extraction step, a final purification step with the High Pure RNA isolation kit (Roche Applied Biosystem) and DNAse I treatments [twice with the DNAse I provided in the kit and once with the reagents from a Turbo DNA-free kit (Applied Biosystems)]. The lack of DNA contamination was checked by PCR on each RNA sample. The RNA integrity was controlled on an agarose gel.

### Quantitative Real-Time PCR

The relative expression levels of the *afeI, afeR, zwf*, AFE_0233, and AFE_1339 genes were compared to that of the 16S rRNA *rrs* gene used as a reference standard by quantitative real-time PCR. RNAs were extracted from planktonic cells grown on S^0^ prills after 2, 3, and 4 days of growth and from sessile cells after 3 days of growth on sulfur prills, as described below. The real-time PCR analysis was performed on a CFX96 real-time PCR detection system with the C1000^TM^ thermal cycler (BioRad) with the “SsoFast EvaGreen Supermix 2X” kit (Bio-Rad) following the manufacturer’s instructions and as described in (Slyemi et al., 2013). The results were analyzed with the Bio-Rad CFX Manager Software 3.0. The real-time quantitative PCR experiments were performed on RNA extracted from at least three independent cultures and duplicated for each RNA preparation with the oligonucleotides listed in Supplementary Table S1. The calculated threshold cycle (Ct) for each gene was normalized to the Ct of the *rrs* gene. The results are expressed in arbitrary units.

### Microarray Construction: Oligonucleotide Design and Arraying

The complete genome (gene annotations and sequences) of *A. ferrooxidans* ATCC 23270^T^ chromosome was downloaded from the NCBI ftp site^1^. The *OliD* program (Talla et al., 2003) was used to design oligonucleotide probe sequences matching defined criteria. An effort was placed to design oligonucleotide probes of similar lengths, with the aim to reduce cross-hybridization between related sequences. Most oligonucleotides are 55 nt long with predicted melting temperatures between 80–100°C in standard hybridization buffer (G + C contents between 30 and 70%). Oligonucleotides were selected such as to avoid self-complementary structures at 65–70°C, and cross-hybridization with the rest of the genome, and were positioned less than 1500 bp upstream of the stop codon of the CDS. The program successfully designed specific oligonucleotide probes for 3044 protein encoding genes, representing 96.7% of the total number of genes. Due to the high similarity with other sequenced regions of the ATCC 23270^T^ genome, 103 genes (3.3%) failed to be represented by a specific oligonucleotide probe. When possible, each gene was represented by two distinct oligonucleotide probes separated by a minimum of 100 nucleotides. A total of 6294 probes from 3147 genes were thus designed. The probes were spotted twice on slides using the Agilent technology^2^. The array design, the experimental design, and the data for all hybridizations are available in Array Express database under accession numbers A-MTAB-592 and E-MTAB-4896.

### Transcriptome Assay

Twelve independent hybridizations using total RNA obtained from three different cultures grown without or with 5 μM of tetrazole **9c** were performed on Agilent microarrays. Total RNA was used for the synthesis of cDNA fluorescent labeled with Cy^®^3 and Cy^®^5 as previously described (Quatrini et al., 2006, 2009). Microarray hybridizations were performed at 42°C for 16 h in a microarray hybridization chamber (Agilent G2534A) following the manufacturer’s instruction. Slides were washed in washing buffer serial dilutions. Arrays were scanned for the Cy^®^3 and Cy^®^5 fluorescent signals using an Axion 4400A scanner (Molecular Devices). The data were analyzed with the image quantification software package GenePix Pro 6.0 (Axon Instruments, Inc.) as previously described (Quatrini et al., 2006, 2009). Each gene expression ratio was calculated from 12 values calculated from three biological and four technical replicates and normalized using Acuity 4.0 package (Molecular Devices). Only the four best hybridizations (in term of reproducibility) out of the six were taken into account. Genes with weak expression (median intensity <250) were discarded. A onefold deviation from the 1:1 hybridization (log_2_) ratio (corresponding to twofold change) was taken as indicative of differential gene expression in the conditions analyzed. The values of one Sample *t*-test – Benjamini–Hochberg (Adv) ≤0.05 (corresponding to 95% confidence) for at least one oligonucleotide were considered statistically significant. Only the genes filling the conditions described above were analyzed. Hierarchical cluster analysis (Pearson correlation, average linkage) was performed using Genesis software suit (Peterson et al., 2001).

### Bioinformatic Analysis

Bioinformatic analyses were performed with the tools available in the MaGe annotation platform^3^ (Vallenet et al., 2013).

### General Biochemical Procedures

The protein concentration was determined by the modified Bradford method (Bio-Rad protein assay). The purity of the preparation was checked by 12.5% SDS-PAGE stained with Coomassie blue and by immunodetection with antibodies directed against the hexa-histidine tag using a SuperSignal West Hisprobe kit (Thermo Scientific) following the manufacturer’s instructions.

### Cloning and Overexpression of *AfeR*

To produce wild-type AfeR fused to a hexa-histidine tag at the C-terminus, the DNA fragment corresponding to the AfeR peptide was amplified by PCR with the AFERC1 and AFERC2 oligonucleotides (Supplementary Table S1). The amplified product was digested with *Hin*dIII and *Xho*I and cloned into pET21 to give pET21-AfeR-Histag plasmid. Cloning was done in *E. coli* TG1 strain. The construction was checked by nucleotide sequencing with the petT7 and T7ter oligonucleotides (Supplementary Table S1). The recombinant plasmid was then introduced into *E. coli* Rosetta (DE3)/pLysS strain.

The Rosetta (DE3)/pLysS strain carrying pET21-AfeR-Histag was grown at 37°C with 100 μg ml^-1^ ampicillin and 34 μg ml^-1^ chloramphenicol to an OD_600_ of 0.6. Ampicillin (100 μg ml^-1^) and 3-hydroxy-C14-AHL (Gonzalez et al., 2013) to a final concentration of 1 μM were then added. Cells were grown 30 min at 30°C. At this stage, 0.4 mM IPTG was added and the culture was grown for a further 3 h at 30°C. The cells were harvested by centrifugation and stored at -80°C until use.

### Production of His-Tagged Recombinant AfeR Protein

To lyse the cells, the cell pellet previously resuspended in lysis solution [50 mM Tris-HCl pH 7.4, 300 mM NaCl, 5 mM imidazole, 2% Tween-20, 1 mM phenylmethylsulfonyl fluoride (PMSF), 0.1 mg ml^-1^ DNase, 0.1 mg ml^-1^ lysosyme, and 5 μM 3-hydroxy-C14-AHL] was incubated 30 min at 4°C with gentle shaking and then sonicated. Inclusion bodies, unbroken cells, and cellular debris were removed by centrifugation at 13,000 rpm for 30 min at 4°C. The pellet was dissolved with 4 M urea in 40 mM sodium phosphate pH 7.4, 300 mM NaCl, 1 mM PMSF, 5 μM 3-hydroxy-C14-AHL, and 0.1 mg ml^-1^ DNase, kept on ice for 30 min with gentle stirring, and then centrifuged at 13,000 rpm for 20 min at 4°C. The supernatant, corresponding to the solubilized inclusion bodies, was filtered through a 0.45 μm membrane before loading onto a cobalt column (HisTrap^TM^ Talon^®^; GE Healthcare) according to the manufacturer’s instructions. The fractions were eluted with 5, 50, 150, 250, and 500 mM imidazole, in 40 mM sodium phosphate pH 7.4, 300 mM NaCl, 1 mM PMSF, 4 M urea, and 25 μM 3-hydroxy-C14-AHL buffer. The 150 mM fractions containing the recombinant AfeR-His tag was dialysed with decreasing urea concentrations (2 M, 0.5 M, 0 M) in 50 mM HEPES pH 8, 150 mM NaCl, 10 mM DTT, and 5 μM 3-hydroxy-C14-AHL. These fractions were kept at 4°C until use.

### Electrophoretic Mobility Shift Assays (EMSA)

DNA substrates for band shift assays were produced by PCR amplification with PrimeSTAR Max DNA Polymerase (Clontech) using 5′ Cy5-labeled reverse oligonucleotide (Sigma; Supplementary Table S1). The Cy5 labeled DNA (2.3 ng) was incubated in a total volume of 10 μl with increasing concentrations of the enriched recombinant AfeR-Histag preparation as indicated in the Figure. The binding reaction contained 20 mM Tris-HCl pH 8, 50 mM KCl, 1 mM DTT, 0.05 % Nonidet P40, 1 mM EDTA, 10 % glycerol, 5 μM 3-hydroxy-C14-AHL, 30 ng μl^-1^ herring sperm, and bovine serum albumin 100 μg ml^-1^. After 30 min at room temperature, the reaction mixtures were separated by electrophoresis on a 6% native polyacrylamide gel previously prerun 5 min and run for 1–2 h more in 25 mM Tris-HCl pH 8.3, 0.19 M glycine, 1 mM EDTA, 200 μM spermidine at 30 mA at 4°C. The gel was then scanned using a 635 nm laser and a LPR filter (FLA5100, Fujifilm).

## Results and Discussion

To develop biological strategies for improving biomining activities and preventing environmental damages caused by AM/RD, it is well documented that mineral colonization by acidophilic bacteria such as *Acidithiobacillus* species is a key step to decipher (Rohwerder and Sand, 2007). If synthesis of specific exopolysaccharides rich in α-mannopyranosyl and α-glucopyranosyl sugar residues has been revealed by fluorescently labeled lectin Concanavalin A within 1 day for EPS (extracellular polymeric substances)-deficient ferrous-iron grown cells after transfer to cultures with pyrite as sole nutrient (Bellenberg et al., 2014), a clear understanding of the molecular cascade involved in exopolysaccharide production and biofilm formation by *Acidithiobacillus* species is actually missing. However, as a molecular relationship between QS and cell adhesion has been clearly established in *A. ferrooxidans* (Gonzalez et al., 2013) and it has to be pointed out that the canonical QS systems are missing in *Acidithiobacillus* species that can oxidize only RISC (Valdés et al., 2008), the iron-oxidizing species such as *A. ferrooxidans* as primary colonizers are now considered fundamental players for mineral colonization by the bioleaching community. Therefore, to address the question of how *A. ferrooxidans* regulates the physiological processes involved in cell adhesion, EPS production and biofilm formation, we focused on the deciphering of the QS molecular network by using a synthetic QS-activator molecule and DNA array technology.

### The Tetrazolic AHL Analog **9c** Accelerates Cellular Adhesion of *Acidithiobacillus ferrooxidans* on Sulfur Coupons

**FIGURE 1:**
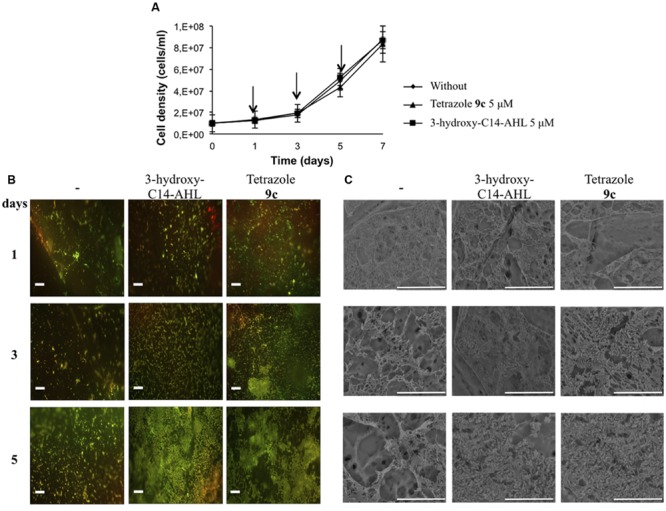
**Effect of tetrazole **9c** on biofilm formation. (A)** Growth curves in the absence or the presence of 5 μM 3-hydroxy-C14-AHL or tetrazole **9c**. Arrows indicate when aliquots were sampled for microscopy analysis. **(B)** Fluorescence microscopy of sulfur coupons after 1, 3, or 5 days of *Acidithiobacillus ferrooxidans* ATCC 23270^T^ cells grown in the absence (-) or the presence of 5 μM 3-hydroxy-C14-AHL or tetrazole **9c**. Cells were stained with fluochrome Syto9. **(C)** Electron microscopy of sulfur coupons treated as above. White bars represent 20 μm.

To further investigate the molecular mechanisms underlying this pathway, we first challenged the identification of synthetic AHL analogs capable to induce better *A. ferrooxidans* cell adhesion than natural AIs previously tested (Gonzalez et al., 2013). Thus, a tetrazolic derivative that displays a much higher affinity to the LuxR protein than the natural AI and acts as a superagonist of AHL signaling molecules (Sabbah et al., 2012) was tested. Its effect on biofilm formation by *A. ferrooxidans* was compared to the natural AI 3-hydroxy-C14-AHL (**Figure 1**). Growth curves revealed that both tetrazolic AHL analog and 3-hydroxy-C14-AHL have no effect on *A. ferrooxidans* growth compared to the control in the absence of exogenous AHL (**Figure 1A**). Fluorescence (**Figure 1B**) and SEM (**Figure 1C**) clearly indicated that tetrazole **9c** also promoted cell adhesion. Moreover, confirming in the *A. ferrooxidans* model the superagonistic behavior of tetrazole **9c** previously found in *V. fisheri* (Sabbah et al., 2012), the results obtained on day 3 strongly suggest that tetrazole **9c** is biologically more efficient than the natural AI 3-hydroxy-C14-AHL in promoting biofilm formation (**Figure 1C**).

### The QS System is Triggered after 3 days in the Presence of the Tetrazolic AHL Analog **9c** in Planktonic Cells

The results presented in **Figure 1** suggest that QS was triggered by 5 μM tetrazole **9c** between 2 and 3 days of growth versus 4–5 days in the absence of this AHL analog. To assess whether the tetrazole **9c** indeed switched on QS system by inducing the transcription of the genes known to be involved in QS response (Farah et al., 2005), i.e. *afeI* (AFE_1999) and *afeR* (AFE_1997), the transcription of these genes was analyzed by quantitative real-time PCR after 2, 3, and 4 days of growth in the presence or the absence of 5μM tetrazole **9c**. The results indicated that *afeR* expression was constitutively expressed under the conditions analyzed, while *afeI* was induced by tetrazole **9c** from the third day of growth in planktonic cells (**Table 1**).

**Table 1:** Quantitative real-time PCR expression data for *afeI, afeR, zwf*, AFE_0233 (glycosyl transferase), and AFE_1339 (putative polysaccharide export protein) genes from *Acidithiobacillus ferrooxidans* ATCC 23270^T^ planktonic cells grown with sulfur prills in the presence or the absence of 5 μM tetrazole **9c** after 2, 3, and 4 days of growth.

Gene or locus name	Growth condition	Day of growth	Gene mRNA/*rrs* ±*SD*^a^
*afeI* (AFE_1999)	DMSO	2	1 ± 0
	Tetrazole **9c**	2	1.69 ± 0.15
	DMSO	3	4.49 ± 0.58
	Tetrazole **9c**	3	12.75 ± 1.66
	DMSO	4	5.19 ± 6.95
	Tetrazole **9c**	4	49.35 ± 5.72
*afeR* (AFE_1997)	DMSO	2	1 ± 0
	Tetrazole **9c**	2	1.58 ± 0.19
	DMSO	3	1.59 ± 0.19
	Tetrazole **9c**	3	1.21 ± 0.08
	DMSO	4	1.64 ± 0.11
	Tetrazole **9c**	4	1.78 ± 0.21
*zwf* (AFE_2025)	DMSO	2	1 ± 0
	Tetrazole **9c**	2	1.43 ± 0.12
	DMSO	3	2.70 ± 1.11
	Tetrazole **9c**	3	1.66 ± 0.16
	DMSO	4	2.82 ± 0.32
	Tetrazole **9c**	4	3.90 ± 0.58
AFE_0233	DMSO	2	1 ± 0
	Tetrazole **9c**	2	1.04 ± 0.01
	DMSO	3	1.13 ± 0.13
	Tetrazole **9c**	3	1.00 ± 0.13
	DMSO	4	1.14 ± 0.17
	Tetrazole **9c**	4	0.91 ± 0.02
AFE_1339	DMSO	2	1 ± 0
	Tetrazole **9c**	2	1.72 ± 0.02
	DMSO	3	1.58 ± 0.32
	Tetrazole **9c**	3	1.62 ± 0.32
	DMSO	4	1.56 ± 0.11
	Tetrazole **9c**	4	1.69 ± 0.12

*^a^Values were related to those obtained after 2 days of growth in the absence of tetrazole **9c**.*

Biofilm formation after 3 days was strongly enhanced in cells treated with 5 μM tetrazole **9c** compared to cells from control experiments without agonist (**Figure 1**). Therefore, expression of some genes predicted to be linked to EPS biosynthesis [*zwf* (AFE_2025), AFE_0233, and AFE_1339] was also monitored in planktonic (**Table 1**) and sessile (Supplementary Table S2) cells after 3 days of growth with 5 μM tetrazole **9c**. The gene *zwf* encodes glucose-6-phosphate 1-dehydrogenase that is involved in the intracellular levels of glucose-6P, a precursor of the EPS. AFE_0233 encodes a glycosyl transferase and is located in a gene cluster predicted to encode cell wall constituents (polysaccharides, and lipopolysaccharides). AFE_1339 encodes the putative polysaccharide export protein Wza and is located close to the *gal* operon proposed to be involved in the formation of EPS in iron-grown cells (Barreto et al., 2005). Besides, AfeR-AHL binding sites were predicted in the regulatory region of *zwf*, AFE_0233, and AFE_1339 (Banderas and Guiliani, 2013). Surprisingly, tetrazole **9c** had no effect on AFE_0233, AFE_1339 and *zwf* transcription and only the expression of the *afeI* gene was induced by tetrazole **9c** (**Table 1**; Supplementary Table S2). These data indicate that *afeI*, and not *afeR*, is regulated by QS and suggest either that *zwf*, AFE_0233, and AFE_1339 genes were not regulated by AfeR or that their expression was induced later during biofilm biogenesis.

### QS Regulon in *Acidithiobacillus ferrooxidans* Cells

Quorum sensing response and biofilm formation were obvious within 3 days of growth in the presence of the tetrazolic AHL analog **9c** (**Figure 1**; **Table 1**). Consequently, total RNAs from planktonic and sessile cells of *A. ferrooxidans* ATCC 23270^T^ were isolated from 3-days cultures in the presence or the absence of the superagonist AHL analog **9c**. They were used to probe gene expression using microarrays displaying two specific oligonucleotides for each gene of this bacterium (3147 predicted genes). Only the genes filling the conditions described in the Materials and Methods section were analyzed. It has to be pointed out that the microarray and quantitative real-time PCR data agreed with the constitutive expression of *afeR, zwf*, AFE_0233, and AFE_1339 genes under the conditions tested (**Table 1**; Supplementary Tables S2–S4).

In planktonic cells, a total of 133 genes were differentially expressed, 34 induced and 99 repressed by tetrazole **9c** (Supplementary Table S3). In sessile cells under the same conditions, only eight genes presented significant differences in expression, four induced and four repressed by tetrazole **9c** (Supplementary Table S4). Therefore, 141 genes were QS regulated, which represent 4.5% of the total number of *A. ferrooxidans* gene analyzed in this study (see Materials and Methods). These genes were grouped according to their COG classification. Their percentage relative to all the *A. ferrooxidans* ATCC 23270^T^ genes present in the same COG class is given in **Table 2**. In planktonic cells, mainly the genes involved in inorganic ion transport and metabolism (4.86%), and nucleotide transport and metabolism (3.39%) were induced in the presence of tetrazole **9c**. Mainly those involved in carbohydrate transport and metabolism (11.11%), posttranslational modification, protein turnover, chaperones (8.27%), energy production and conversion (5.76%), cell motility (3.70%), and transcription (2.92%) as well as poorly characterized proteins (11%) were repressed by this AHL analog. In sessile cells, mainly induction by tetrazole **9c** of secondary metabolites biosynthesis, transport and catabolism (1.61%), and signal transduction mechanisms (1.15%) was observed while repression was detected for energy production and conversion genes (1.05%). Only the genes differentially expressed in cells that were cultivated with or without the tetrazolic AHL analog and which have known or reliable predicted function are presented in **Table 3** for the planktonic cells and in **Table 4** for the sessile cells and are discussed below.

**Table 2:** COG classification of the genes differentially expressed in planktonic and sessile cells grown with (+) and without (-) tetrazole **9c**.

Process	COG functional categories	COG class	Planktonic cells^a,b^		Sessile cells^a,b^	
			+	-	+	-
Cellular processes and signaling	Cell cycle control, cell division, chromosome partitioning	D	0.00%	0.00%	0.00%	0.00%
	Cell wall/membrane/envelope biogenesis	M	0.93%	0.93%	0.00%	0.00%
	Cell motility	N	0.00%	**3.70%**	0.00%	0.00%
	Posttranslational modification, protein turnover, chaperones	O	0.00%	**8.27%**	**0.75%**	0.00%
	Signal transduction mechanisms	T	0.00%	**1.15%**	**1.15%**	0.00%
	Intracellular trafficking, secretion, and vesicular transport	U	**1.89%**	0.94%	0.00%	0.00%
	Defense mechanisms	V	0.00%	0.00%	0.00%	0.00%
	Extracellular structures	W	0.00%	0.00%	0.00%	0.00%
Information storage and processing	RNA processing and modification	A	0.00%	0.00%	0.00%	0.00%
	Chromatin structure and dynamics	B	0.00%	0.00%	0.00%	0.00%
	Translation, ribosomal structure, and biogenesis	J	**1.89%**	0.00%	0.00%	0.00%
	Transcription	K	0.00%	**2.92%**	0.00%	0.00%
	Replication, recombination, and repair	L	0.00%	**1.35%**	0.00%	0.00%
Metabolism	Energy production and conversion	C	2.09%	**5.76%**	0.00%	**1.05%**
	Amino acid transport and metabolism	E	1.01%	1.52%	0.00%	0.00%
	Nucleotide transport and metabolism	F	**3.39%**	0.00%	0.00%	0.00%
	Carbohydrate transport and metabolism	G	1.59%	**11.11%**	0.00%	0.00%
	Coenzyme transport and metabolism	H	0.00%	**1.74%**	0.00%	0.00%
	Lipid transport and metabolism	I	0.00%	**1.41%**	0.00%	0.00%
	Inorganic ion transport and metabolism	P	**4.86%**	1.62%	0.54%	0.54%
	Secondary metabolites biosynthesis, transport, and catabolism	Q	0.00%	**1.61%**	**1.61%**	0.00%
Poorly characterized	General function prediction only	R	0.31%	**4.97%**	**0.62%**	0.31%
	Function unknown	S	0.00%	**6.03%**	0.00%	0.00%

*^a^The numbers represent the percentage relative to all the *A. ferrooxidans* ATCC 23270^T^ genes present in this COG class. ^b^Bold numbers are discussed in the text.*

**Table 3:** Microarray expression data for genes with known or predicted function differentially expressed in planktonic cells in the presence of tetrazole 9c.

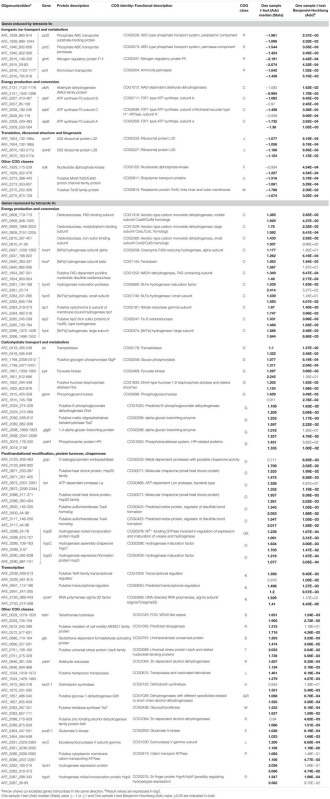

**Table 4:** Microarray expression data for genes with known or predicted function differentially expressed in sessile cells in the presence of tetrazole 9c.

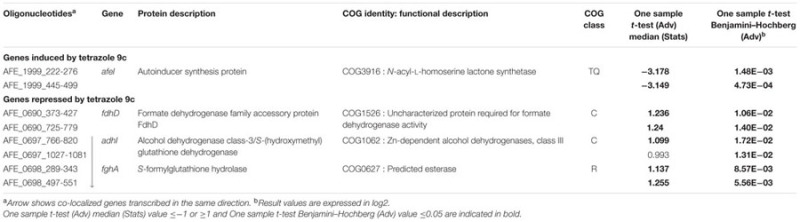

### Genes Differentially Expressed in the Presence of Tetrazole **9c** in Planktonic Cells

In planktonic cells, tetrazole **9c** modified the expression of a number of genes related to biofilm formation, few being induced and several repressed. Among the induced genes, those involved in inorganic ion transport and energy conversion were mainly found. Not surprisingly, genes involved in the transport of phosphate [*pstS* (AFE_1939) and *pstC* (AFE_1940)] and ammonium [*glnK* (AFE_2915) and *amt* (AFE_2916)] were upregulated. The phosphate specific transport (Pst) system is known to be important in biofilm formation in a number of bacteria [see (O’May et al., 2009; Heindl et al., 2014) and references therein] including *Leptospirillum ferrooxidans* (Moreno-Paz et al., 2010) and *A. ferrooxidans* (Vera et al., 2013), in which phosphate metabolism was early linked to QS regulatory pathway (Farah et al., 2005). Deep cDNA sequencing experiments also revealed that several genes related to ammonium metabolism (*amt-1, amt-2*, and *glnK-1*) were upregulated in *A. ferrooxidans* planktonic cells induced by hydroxyl-C14-AHL compared to not induced (unpublished data). Biofilm formation occurs also in response to the availability of nutrients supplied by the ammonium transporter (AFE_2916) which expression is regulated by GlnK (AFE_2915), as shown recently in *Streptococcus mutans* (Ardin et al., 2014). This might anticipate gradient of inorganic ions within and around microbial biofilm. The other gene class that was induced by tetrazole **9c** in planktonic cells is involved in energy production and conversion, in particular the genes *atpBEF* (AFE_3207–3209) encoding the membrane-embedded proton channel F0 of the ATPase. This upregulation could allow more protons to pass through the ATP synthase complex generating a proton motive force (PMF) rather than ATP. PMF is required not only for early biofilm formation (Saville et al., 2011), but also in influx and eﬄux involved in QS since PMF inhibition enhances the intracellular accumulation of AHL leading to decrease in biofilm formation (Ikonomidis et al., 2008; Varga et al., 2012). Along the same lines, genes encoding a putative MolA/TolQ/ExbB proton channel family protein (AFE_2273) and TonB family protein (AFE_2275) were upregulated in the presence of the tetrazole **9c** and could contribute to PMF-dependent import through the outer membrane of substrates necessary for QS and/or early EPS synthesis. Another interesting gene that was more expressed in the presence of the tetrazolic AHL analog in planktonic cells is *ndk* (AFE_1929) encoding a nucleoside diphosphate kinase. A *ndk* knockout mutant of *Pseudomonas aeruginosa* was shown to be deficient in polysaccharide synthesis (Kapatral et al., 2000), because it was unable to provide GTP necessary for the incorporation of mannuronate in alginate. It is therefore possible that nucleotide triphosphates are required in an early step of *A. ferrooxidans* EPS biosynthesis.

The genes that were repressed in the presence of the tetrazolic AHL analog in planktonic cells were mainly involved in energy production and conversion, carbohydrate transport and metabolism, posttranslational modification, protein turnover, chaperones, and transcription. Most of the energy production and conversion class genes encoded two out of the four hydrogenases described in *A. ferrooxidans*. One is a group one membrane-bound respiratory enzyme enabling the cell to use H_2_ as an energy source [*hynS* (AFE_3283) and *hynL* (AFE_3286)]. The genes encoding this hydrogenase physiological partners [*isp1* (AFE_3284) and *isp2* (AFE_3285)] and biogenesis machinery [*hynD* (AFE_3281), *hynH* (AFE_3282), *hynL* (AFE_3286), *hypA* (AFE_3287), *hypB* (AFE_3288), *hypC* (AFE_3289), and *hypD* (AFE_3290)] were also repressed under this condition. The second hydrogenase is a sulfhydrogenase, a group 3b cytoplasmic hydrogenase [*hoxH* (AFE_0937) and *hoxF* (AFE_0940)], with both hydrogenase and sulfur reductase activities, likely serving as an electron sink under highly reducing conditions by recycling redox cofactors using either protons or polysulfides as the electron acceptor. It is worth mentioning that, in different bacteria, some hydrogenases were shown to be upregulated in sessile cells, others in planktonic cells (Caffrey et al., 2008; Clark et al., 2012; Kassem et al., 2012). Our data suggest that the groups 1 and 3 hydrogenases of *A. ferrooxidans* are specific to the non-attached cells.

**FIGURE 2:**
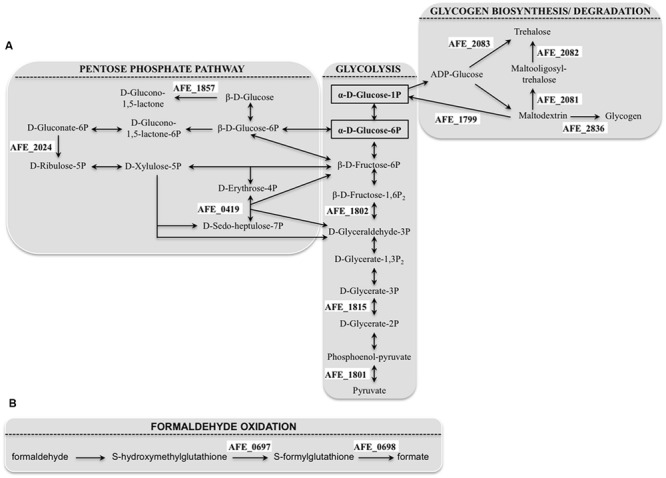
***Acidithiobacillus ferrooxidans* pathways showing gene repression by tetrazole **9c**. (**A**)** Genes involved in carbon flow that were downregulated in planktonic cells (for clarity, only the connections of the pathways discussed in the text have been shown). **(B)** Genes involved in formaldehyde oxidation that were downregulated in sessile cells. The precursors of the EPS (extracellular polymeric substances) building blocks are surrounded by a black rectangle. The genes that were repressed by tetrazole **9c** are indicated in a white background.

The number of genes belonging to the carbohydrate transport and metabolism class that were differentially expressed with/without tetrazole **9c** agrees with an alteration in the carbon flow when planktonic cells switched to sessile state. It has to be pointed out that all these genes were downregulated in the presence of the tetrazole **9c**. Three pathways seemed to be affected: the glycolysis [*pyk* (AFE_1801), AFE_1802, *gpmL* (AFE_1815)], the pentose phosphate pathway [*tal* (AFE_0419), AFE_1857, and AFE_2024] and the glycogen biosynthesis/degradation pathway [AFE_1799, AFE_2082, AFE_2083, and *glgB* (AFE_2836)]. In the case of glycolysis, this could mean that the pathway was directed toward β-D-fructose-1,6-bisphosphate, β-D- fructose-6-phosphate, α-D-glucose-6-phosphate, and α-D-glucose-1-phosphate production (**Figure 2A**). Similarly, in the pentose phosphate pathway, the repression would lead toward β-D-glucose, β-D-glucose-6-phosphate and β-D-fructose-6-phosphate direction and therefore to α-D-glucose-6-phosphate and α-D-glucose-1-phosphate accumulation (**Figure 2A**). Noteworthy, α-D-glucose-6-phosphate and α-D-glucose-1-phosphate are the precursors of UDP glucose, UDP-galactose, dTDP-rhamnose and GDP-mannose, which are the building blocks in EPS biosynthesis (Quatrini et al., 2007). Another interesting results was the repression of three genes predicted to be involved in trehalose synthesis [*treT* (AFE_2083), *treZ* (AFE_2082) and *treY* (AFE_2081)] by tetrazole **9c**. In the first case, α-D-glucose-1-phosphate consumption will be prevented, in agreement with the data presented above, and, in addition, maltodextrin synthesis will be favored. In the second case, maltodextrin consumption will be avoided (**Figure 2B**). Notably, maltodextrin has been shown to increase *E. coli* adhesion (Nickerson and McDonald, 2012). Along the same lines, genes involved in maltodextrin consumption [AFE_1799 and *glgB* (AFE_2836)] were repressed in the presence of tetrazole **9c** (**Figure 2B**). Therefore, in planktonic cells, it appears that tetrazole **9c** directed the carbon flow toward adhesion (maltodextrin), EPS precursor biosynthesis (α-D-glucose-6-phosphate, α-D-glucose-1-phosphate) and therefore biofilm formation.

In a number of microorganisms, including *L. ferrooxidans*, heat shock chaperones (Moreno-Paz et al., 2010; Singh et al., 2012; Becherelli et al., 2013; Grudniak et al., 2013) and proteases (Doern et al., 2009; Moreno-Paz et al., 2010; Singh et al., 2012; Yepes et al., 2012), in particular O-sialoglycoprotein endopeptidase (Wickstrom et al., 2013), have been shown to be required in sessile cells for biofilm development. Furthermore, the *uspA* gene, encoding an universal stress protein, is necessary for optimal biofilm formation in *Porphyromonas gingivalis* (Chen et al., 2006). In *A. ferrooxidans*, tetrazole **9c** repressed the genes encoding the heat shock response RNA polymerase σ32 factor [*rpoH* (AFE2750)], Hsp20 family heat shock proteins (AFE_0871 and 2086), a putative universal stress protein (AFE_0751), as well as protease [*lon* (AFE_0872)] and O-sialoglycoprotein endopeptidase [*gcp* (AFE_0123)] in planktonic cells, indicating that these proteins are not required at the early step of biofilm biogenesis. Interestingly, *bioD* (AFE_1675) was repressed in the presence of the tetrazolic analog. This gene encodes dethiobiotin synthetase involved in biotin synthesis from 7-keto-8-aminopelargonate. This pathway consumes *S*-adenosyl-L-methionine (Streit and Entcheva, 2003). The down-regulation in the presence of tetrazole **9c** of this gene could save this substrate that is required for AHL biosynthesis. Another important data is the repression of *proB* (AFE_2464) in the presence of tetrazole **9c**. The *proB* gene encodes glutamate-5-kinase and its repression could lead to glutamate accumulation. Glutamate metabolism has been reported to be essential for biofilm formation. Amino acid levels in general increased in biofilm cells and are used as precursors for energy production with gluconeogenesis (Yeom et al., 2013). In harsh environments, such as acidic conditions, a high demand for amino acids as substrates for energy production may indeed exist in biofilms. Very recently, it has been proposed that amino acids, including glutamate, may also have another role as a signal for biofilm maturation and eventual disassembly (Wong et al., 2015). Finally, two genes encoding transcriptional regulators (AFE_2209 and AFE_2641) were repressed in planktonic cells in the presence of tetrazolic AHL analog. Therefore, we cannot exclude the possibility that genes differentially expressed in the presence of this superagonist AHL analog were indirectly regulated by one of these regulators rather than directly by the AfeR/AfeI QS system. It is noteworthy that members of the TetR-protein family, as is the case for AFE_2209, have been directly involved in the regulation of cellular processes and in particular the QS in different Gram-negative species (Cuthbertson and Nodwell, 2013; Longo et al., 2013).

To summarize, in planktonic cells, tetrazole **9c** led to the induction of genes encoding (i) proton channel proteins to allow PMF energized transport system of AHL and substrates required for EPS synthesis, (ii) an enzyme required in an early step of polysaccharide synthesis, and (iii) transport system to anticipate phosphate and ammonium gradients within the biofilm. On the other hand, it repressed genes involved in (i) biofilm maturation (heat-shock proteins and chaperone encoding genes), (ii) biotin synthesis to prevent the consumption of *S*-adenosyl-L-methionine required for AHL biosynthesis, (iii) glutamate conversion to proline to use it as an energy source and/or as a signal for biofilm maturation, and (iv) carbohydrate metabolism to redirect the carbon flow toward proteins necessary for adhesion and EPS precursor biosynthesis. It seems therefore reasonable to conclude that tetrazole **9c** reprograms planktonic cells toward early biofilm formation.

### Genes Differentially Expressed in the Presence of Tetrazole **9c** in Sessile Cells

In sessile cells, only four genes, encoding proteins with known or predicted functions, presented significant differences in expression. Not surprisingly, the gene with the highest fold difference was *afeI* (AFE_1999) encoding the AHL synthase, with at least an eight-fold expression increase in the presence of tetrazole **9c** indicating that indeed the QS was triggered. The three other genes *fdhD* (AFE_0690), *adhI* (AFE_0697), and *fghA* (AFE_0698) encoding a putative formate dehydrogenase family accessory protein FdhD, a *S*-(hydroxymethyl) glutathione dehydrogenase, and a *S*-formylglutathione hydrolase, respectively, are involved in formaldehyde oxidation to formate (**Figure 2B**). Their repression could lead to the accumulation of formaldehyde, shown to lead to higher biofilm density in a biofilm reactor (Ong et al., 2006). Another not exclusive possibility is that this system is to prevent formate formation that could acidify *A. ferroxidans* cytoplasm and lead to cell death.

Surprisingly, only three genes differentially expressed in the presence of tetrazole **9c** (Supplementary Tables S3 and S4) have the AfeR binding site inferred from bioinformatic prediction (Farah et al., 2005; Banderas and Guiliani, 2013): AFE_0582 and AFE_1998 encoding hypothetical proteins as well as *afeI* (AFE_1999). This could be due to an indirect regulation through a regulator whose expression is controlled by QS. However, the two genes encoding a transcription regulator whose expression was downregulated in the presence of tetrazole **9c** (AFE_2209 and AFE_2641) do not exhibit this predicted AfeR binding site. On the other hand, three genes [*zwf* (AFE_2025), AFE_0233, and AFE_1339] in which this site was predicted, are constitutively expressed in the conditions analyzed. Therefore, another possibility is that a different transcriptional regulator than AfeR binds to the proposed AfeR binding site. All in all, the QS regulon of *A. ferrooxidans* seems to involve a complex regulatory cascade.

### AfeR Binds Specifically to the *afeI* Regulatory Region

**FIGURE 3:**
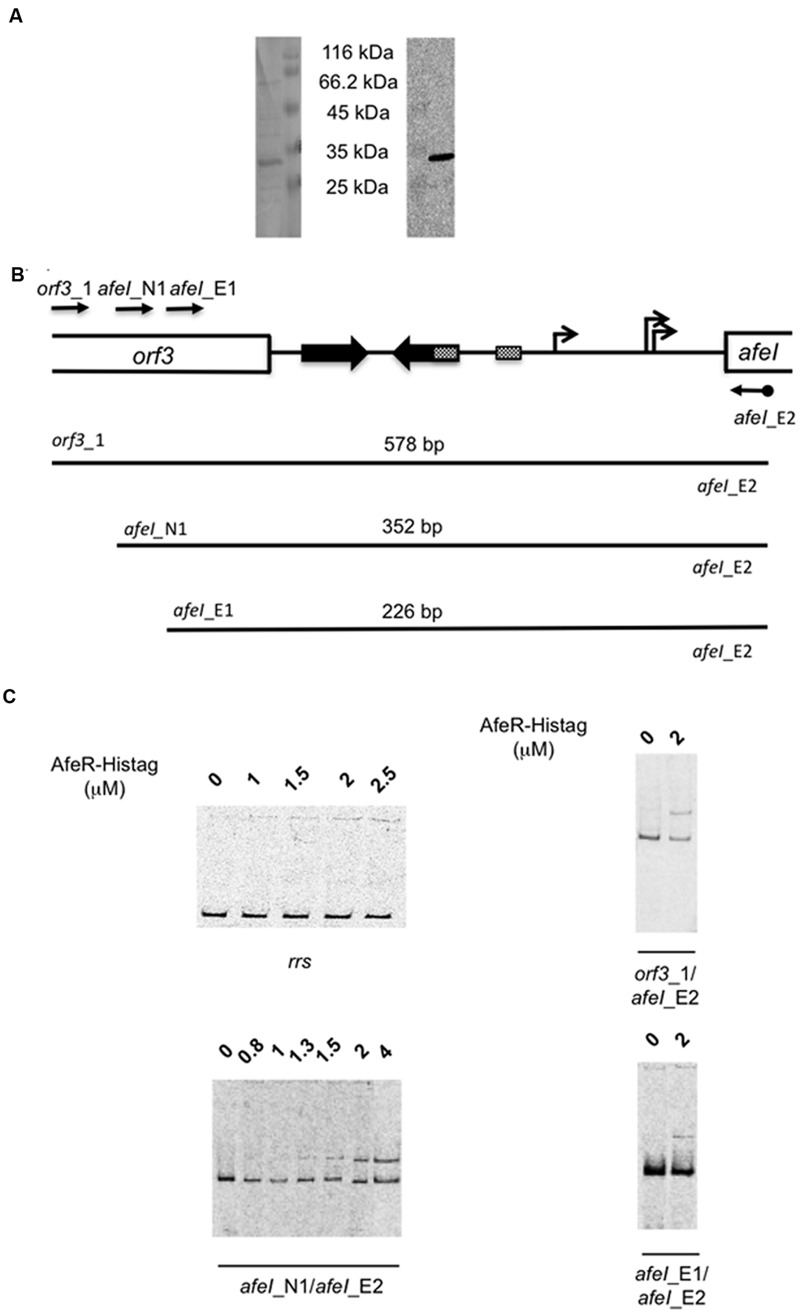
**Production of the recombinant AfeR-Histag in *Escherichia coli* and its binding on the *afeI* regulatory region. (**A**)** Coomassie brilliant blue stained SDS-PAGE (left) and Western immunoblot with antisera raised against the hexahistidine tag (right). The size of the unstained protein molecular weight marker standards (Euromedex) is indicated. **(B)** Schematic representation of the *afeI* locus with the DNA fragments analysed. **(C)** Gel mobility shift assays with an internal DNA fragment of the *rrs* gene of *Thiomonas arsenitoxydans* (left upper part) and the DNA fragments depicted in **(B)**.

To check that the *afeI* induction in the presence of tetrazole **9c** observed by transcriptomic data was mediated by the QS regulator AfeR, we have produced AfeR in *E. coli* and analyzed its binding to the regulatory region of the *afeI* gene. AfeR with a hexa-histidine tag fused to its C terminus (AfeR-Histag) was mainly found in the inclusion bodies, even when the 3-hydroxy-C14-AHL (Gonzalez et al., 2013) was added at the induction time. The recombinant AfeR-Histag produced in the presence of 3-hydroxy-C14-AHL was purified on an affinity cobalt column. As shown in **Figure 3A**, a major band of the expected mass (theoretical molecular mass: 27,876 Da including one molecule of 3-hydroxy-C14-AHL) was visualized on Coomassie blue-stained SDS-polyacrylamide gels. This same protein was recognized by anti-hexahistidine tag antibodies (**Figure 3A**) strongly suggesting that it was AfeR-Histag. The analysis by MALDI-TOF mass spectrometry of this protein digested with Trypsin after reduction by DTT and alkylation by iodoacetamide confirmed that it was AfeR-Histag (54% sequence coverage).

Binding of AfeR-Histag to the regulatory region of *afeI* was analyzed by EMSA in the presence of 3-hydroxy-C14-AHL. A retarded band was detected with 1.3 μM AfeR-Histag and higher concentrations (**Figure 3C**) with DNA fragments encompassing the palindromic sequence predicted to be the AfeR binding site (Farah et al., 2005; Banderas and Guiliani, 2013) in the *afeI* regulatory region (**Figure 3B**). This binding was specific to this region since no binding was observed on an internal fragment of the *rrs* gene of *Thiomonas arsenitoxydans* (**Figure 3C**). These results indicate that AfeR-Histag binds to the regulatory region of *afeI* in the presence of 3-hydroxy-C14-AHL, in agreement with the induction of this gene in the presence of tetrazole superagonist AHL analog **9c**. Since AfeR was constitutively expressed under the conditions analyzed (i.e., with or without tetrazole **9c**), these results suggest that the binding of 3-hydroxy-C14-AHL to AfeR induces a conformational change allowing its specific binding to the target DNA, as it has been proposed for several members of LuxR-like protein family (Choi and Greenberg, 1991).

## Conclusion

The exogenous use of tetrazole superagonist AHL analog **9c** allowed the first overview of the QS regulon of *A. ferrooxidans*, an acidophilic bacterial species involved in bioleaching processes. This study gave some insights into the molecular chain reactions involved in the first steps of mineral adherence and colonization of this bacterium. As expected, tetrazole **9c** activates the positive feedback previously reported (Rivas et al., 2005) by inducing the transcription of *afeI* gene, likely through its binding to the transcriptional regulator AfeR, and therefore its activation, as early as the third day of growth.

The data obtained from planktonic cells revealed that tetrazole **9c** triggers the QS system to drive gene expression toward sessile state by reprogramming some cellular processes. These mainly include: (i) induction of the genes encoding the F0-ATPase subunit leading to the PMF allowing AHL eﬄux and influx, (ii) repression of several genes involved in carbohydrate metabolism to orientate carbon flow to maltodextrin and EPS building block precursor synthesis for adhesion and biofilm formation, respectively; (iii) induction of phosphate and ammonium transporters to anticipate inorganic ion gradient within and around the biofilm structure. Whereas QS and c-di-GMP pathway have been linked in different bacterial species (Waters et al., 2008; Zhang, 2010; Kozlova et al., 2011; Suppiger et al., 2016), it is noteworthy that no change in the transcriptional profiling of the seven genes related to the c-di-GMP pathway in *A. ferrooxidans* (Ruiz et al., 2012; Castro et al., 2015) has been observed in the presence of tetrazole **9c.** This result indicates that QS does not modulate c-di-GMP signaling in this Gram-negative species. Finally, the high transcription level of *afeI* gene in sessile cells observed after 3 days of growth lead not only to *A. ferrooxidans* biofilm stabilization but also to the synthesis of a large spectrum of AHL molecules (Farah et al., 2005; Valenzuela et al., 2017), some of which are sensed by secondary colonizers such as *A. thiooxidans* to form a mixed biofilm (Bellenberg et al., 2014) through a not yet identified non-canonical AHL-binding protein.

## Author contributions

VB and NG conceived and designed the experiments. SM, DM, YD, and ET performed the experiments. VB, SM, NG, and DM analyzed the data. LS and YQ performed the chemical synthesis. NG, VB, YD, LS, YQ, and ET contributed to the reagents/materials/analysis tools. VB, NG, and ET wrote the paper. All authors read and approved the final manuscript.

## Conflict of Interest Statement

The authors declare that the research was conducted in the absence of any commercial or financial relationships that could be construed as a potential conflict of interest.

## Footnotes

1 ftp://ftp.ncbi.nlm.nih.gov/

2 http://www.agilent.com/home

3 https://www.genoscope.cns.fr/agc/microscope/home/

## Supplementary Material

FIGURE S1 **Chemical structure of tetrazole 9c**.
