# Supplementary material for: Insights into the Quorum Sensing Regulon of the Acidophilic Acidithiobacillus ferrooxidans Revealed by Transcriptomic in the Presence of an Acyl Homoserine Lactone Superagonist Analog
Source: Front Microbiol. 2016 Sep 14;7:1365. doi: 10.3389/fmicb.2016.01365 (PMC5021923; doi:10.3389/fmicb.2016.01365)
Supplement: Supplementary file 2 [file Table_1.DOCX]

**Table S1. Oligonucleotides used in this study.**

| **Target DNA** | **Primer** | **Primer sequence** | **Annealing temperature** | **Amplicon size** |
| --- | --- | --- | --- | --- |
| **Real-time PCR** | | | | |
| ***afeI*** | *afeI*_T11 | CCCTTCCAGCTGAAGTTTTACCG | 60°C | 253 bp |
|  | *afeI*_T22 | CCCTTGTGCCAGTCCGTTGAGTA |  |  |
| ***afeR*** | *afeR*_11 | ACGACCTCTTTGGCACTG | 59°C | 222 bp |
|  | *afeR*_22 | CAGATATCAAGCGCAGGAAT |  |  |
| ***zwf*** | *zwf*_1 | CGATATGGAACCGCAATTACA | 60°C | 211 bp |
|  | *zwf*_2 | CGCAGGACTTTGACCTT |  |  |
| **AFE_1339** | AFE1339-1 | TCGTATTGTGGCGGTGGATG | 60°C | 209 bp |
|  | AFE1339-2 | TAAGGCCGACTTGATCCTGT |  |  |
| ***rrs*** | 16S-G | ACACTGGGACTGAGACACGG | 58°C | 277 bp |
|  | 16S-D | ACCGCCTACGCACCCTTTAC |  |  |
| **AFE_0233** | *wcaA*-Fwd | AGGGGAGCGTCAATCGGTAAA | 58°C | 183 bp |
|  | *wcaA*-Rev | ACCCTGGCATCTGGACAAAA |  |  |
| **Expression plasmid construction ^a, b^** | | | | |
| ***afeR*** | *afeR*_C1 | AAA*AAGCTT*GGGAAGGAGGGACATTTGGATC | 50°C | 747 bp |
|  | *afeR*_C2 | AAA*CTCGAG*CGACAGCAACCCGAGCATCG^*^ |  |  |
| **pET21** | petT7 | GTGAGCGGATAACAATTCCCC | 60°C | 966 bp |
|  | T7 ter | CAGCCAACTCAGCTTCCTT |  |  |
| **Electrophoretic mobility shift assays ^c^** | | | | |
| ***orf3*** | *orf3*_1 | GAACGGAATCGCGGTAAA | 58°C^#^ | 578 bp^#^ |
|  | *afeI*_N1 | AAGGTACCTGGGGTGGGACTGGCGGATGGA | 61°C^#^ | 352 bp^#^ |
|  | *afeI* _E1 | GGGTCCCGCCATTACAGCCA | 61°C^#^ | 226 bp^#^ |
| ***afeI*** | AFEI/E2-Cy5 | Cy5-GTAGCCGGCCACCTTCGCATAC |  |  |
| ***rrs*** | 16S-Cy5 | Cy5-CACACCGCCCGTCACACC | 60°C | 120 bp |
|  | 16S rev2 | CCCCAGTCATGAAGCCTACC |  |  |

**a,** restriction sites are shown in italics ; **b,** Shine-Dalgarno sequence is underlined ; **c,** with *afeI*_E2-Cy5.
