# Supplementary material for: Insights into the Quorum Sensing Regulon of the Acidophilic Acidithiobacillus ferrooxidans Revealed by Transcriptomic in the Presence of an Acyl Homoserine Lactone Superagonist Analog
Source: Front Microbiol. 2016 Sep 14;7:1365. doi: 10.3389/fmicb.2016.01365 (PMC5021923; doi:10.3389/fmicb.2016.01365)
Supplement: Supplementary file 3 [file Table_2.DOCX]

**Table S2. Quantitative real-time PCR expression data for *afeI, afeR, zwf*, AFE_0233 (glycosyl transferase) and AFE_1339 (putative polysaccharide export protein) genes from *Acidithiobacillus ferrooxidans* ATCC 23270^T^ adhered cells grown on sulfur prills in the presence or the absence of 5 μM tetrazole 9c after three days of growth.**

| **Gene or**  **locus name** | **Growth**  **condition** | **Gene mRNA/*rrs***  **± SD^a^** |
| --- | --- | --- |
| *afeI* (AFE_1999) | DMSO | 1 ± 0 |
|  | Tetrazole **9c** | 9.05 ± 0.32 |
| *afeR* (AFE_1997) | DMSO | 1 ± 0 |
|  | Tetrazole **9c** | 0.73 ± 0.05 |
| *zwf* (AFE_2025) | DMSO | 1 ± 0 |
|  | Tetrazole **9c** | 0.78 ± 0.01 |
| AFE_0233 | DMSO | 1 ± 0 |
|  | Tetrazole **9c** | 0.69 ± 0.05 |
| AFE_1339 | DMSO | 1 ± 0 |
|  | Tetrazole **9c** | 0.50 ± 0.04 |

**a**, Values were related to those obtained in the absence of tetrazole **9c**.
