# Supplementary material for: Insights into the Quorum Sensing Regulon of the Acidophilic Acidithiobacillus ferrooxidans Revealed by Transcriptomic in the Presence of an Acyl Homoserine Lactone Superagonist Analog
Source: Front Microbiol. 2016 Sep 14;7:1365. doi: 10.3389/fmicb.2016.01365 (PMC5021923; doi:10.3389/fmicb.2016.01365)
Supplement: Supplementary file 5 [file Table_4.PDF]

Table S4. Differentially expressed genes in sessile cells grown with or without tetrazole 9c.

Higher expression **with** tetrazole **9c**:

Median ≤ -1.5

Median ≤ -1 and > - 1.5

Higher expression **without** tetrazole 9

Median ≥1.5

Median ≥1 and < 1.5

	Oligonucleotides[a, b]	gene	protein description	COG identity: function	COG class	One Sample t-Test (Adv) Median (Stats)	One Sample t-Test Benjamini-Hochberg (Adv)[c]
1	AFE_0690_373-427	*fdhD-2*	formate dehydrogenase family accessory protein FdhD	COG1526: Uncharacterized protein required for formate dehydrogenase activity	C	**1,236**	**1,06E-02**
	AFE_0690_725-779					**1,24**	**1,40E-02**
2	AFE_0697_766-820		S-(hydroxymethyl)glutathione dehydrogenase/class III alcohol dehydrog	COG1062: Zn-dependent alcohol dehydrogenases, class III	C	**1,099**	**1,72E-02**
	AFE_0697_1027-1081					0,993	**1,31E-02**
3	AFE_0698_289-343	*fghA*	S-formylglutathione hydrolase	COG0627: Predicted esterase	R	1,137	**8,57E-03**
	AFE_0698_497-551					**1,255**	**5,56E-03**
4	AFE_0707_502-556		hypothetical protein	COG0457: FOG: TPR repeat	R	**-1,025**	**6,31E-03**
	AFE_0707_905-959					-0,916	**1,59E-02**
5	AFE_0709_171-225		hypothetical protein			**-1,194**	**1,48E-03**
	AFE_0709_35-89					**-1,287**	1,63E-01
6	AFE_0710_440-494		iron-sulfur cluster-binding protein, Rieske family	COG4638: Phenylpropionate dioxygenase and related ring-hydroxylating dioxygenases, large terminal subunit; COG0694: Thioredoxin-like proteins and domains	PR	-0,718	**3,48E-02**
	AFE_0710_651-705				O	**-1,069**	5,95E-02
7	AFE_1998_141-195		hypothetical protein			**-2,245**	**1,45E-03**
	AFE_1998_338-392					**-1,98**	**1,84E-03**
8	AFE_1999_222-276	*afeI*	autoinducer synthesis protein	COG3916: N-acyl-L-homoserine lactone synthetase	TQ	**-3,178**	**1,48E-03**
	AFE_1999_445-499					**-3,149**	**4,73E-04**

a, Arrows show co-localized genes transcribed in the same direction. **b**, The genes discussed in the text are highlighted in grey. **c**, Result values are expressed in log2.
